# Drug-disease interaction: Clinical consequences of inflammation on drugs action and disposition

**DOI:** 10.3389/jpps.2023.11137

**Published:** 2023-02-06

**Authors:** Yasmeen El Hajj Abdallah, Sukhman Chahal, Fakhreddin Jamali, Sherif Hanafy Mahmoud

**Affiliations:** Faculty of Pharmacy and Pharmaceutical Sciences, University of Alberta, Edmonton, AB, Canada

**Keywords:** drug-disease interaction, inflammation, drugs action, drugs disposition, drugs

## Abstract

Inflammation is a culprit in many conditions affecting millions of people worldwide. A plethora of studies has revealed that inflammation and inflammatory mediators such as cytokines and chemokines are associated with altered expression and activity of various proteins such as those involved in drug metabolism, specifically cytochrome P450 enzymes (CYPs). Emphasis of most available reports is on the inflammation-induced downregulation of CYPs, subsequently an increase in their substrate concentrations, and the link between the condition and the inflammatory mediators such as interleukin-6 and tumor necrosis factor alpha. However, reports also suggest that inflammation influences expression and/or activity of other proteins such as those involved in the drug-receptor interaction. These multifaced involvements render the clinical consequence of the inflammation unexpected. Such changes are shown in many inflammatory conditions including rheumatoid arthritis, Crohn’s disease, acute respiratory illnesses as well as natural processes such as aging, among others. For example, some commonly used cardiovascular drugs lose their efficacy when patients get afflicted with inflammatory conditions such as rheumatoid arthritis and Crohn’s disease. Interestingly, this is despite increased concentration subsequent to reduced clearance. The observation is attributed to a simultaneous reduction in the expression of target receptor proteins such as the calcium and potassium channel and β-adrenergic receptor as well as the metabolic enzymes. This narrative review summarizes the current understanding and clinical implications of the inflammatory effects on both CYPs and drug-receptor target proteins.

## Introduction

Inflammation is a non-specific process associated with many conditions ranging from mental diseases (1) to pain (2) arthritis, cancer, obesity and old age, among others (3). Through its systemic effects, inflammation influences expression of various active proteins thereby, alters action and disposition of drugs. The effect of inflammation on the proteins involved in drug metabolism is known for decades (4). Surprisingly, however, the knowledge of such effect on other proteins such as those essential for the drug-receptor interactions is not well-known. Thus, the therapeutic inferences of such drug-disease interactions are commonly based on the altered systemic concentration of drugs and not the true pharmacodynamic outcomes. This review provides evidence that mere altered pharmacokinetics do not necessarily amount to corresponding pharmacodynamic changes.

Inflammatory reactions are adaptive and protective responses from the immune system to a variety of stimuli that may pose a risk to the body. To eliminate the pathogen from the host and initiate healing, inflammation yields alterations in homeostasis. The process of inflammation involves a variety of immune cells, which are coordinated by distinct chemical messengers known as cytokines (5). Although when inflammation is acute it is well-coordinated with the immune system, it can evolve into harmful chronic states if it is overstimulated or left unresolved.

Inflammation regulates the immune system by causing innate and adaptive immunity. Innate immunity is the early, non-specific response to pathogens that occurs rapidly and involves antigen presenting cells (APCs), such as phagocytes and dendritic cells, combating pathogens that have breached the skin barrier (6). Subsequently, some surviving APCs migrate into lymph nodes to initiate the adaptive immune process. Adaptive immunity is the slower and more pathogen-specific response where the APCs present the antigen to T lymphocytes (namely T helper cells), which in turn, activate B-cells to create the antigen specific antibodies and memory B cells. Thus, inflammation plays a supportive role in the T lymphocyte activation and memory B cell imprinting process, thereby, contributing to the body’s protection from future insults (6).

Inflammation operates through the immune system using cytokine signaling systems. This process is defined by the cardinal signs of rubor, calor, tumor, and dolor functions *via* activation of pattern-recognition receptors on the innate immune cells with inflammatory stimuli (5, 6).

Vasodilation, extravasation and increased vessel permeability to proteins, enzymes, cytokines, both pro-inflammatory and anti-inflammatory, lead to further recruitment of innate and adaptive immune cells. These cytokines induce the chemotaxis and diapedesis of circulating leukocytes which further release inflammatory mediators and initiate destruction of the foreign antigens (8). This process is often restricted to a particular area affected by injury or infection; however, maladaptive cases may occur, leading to an excess production of inflammatory mediators, leading to further spread of the insult (5).

In a typical inflammatory response, a fine balance exists between T lymphocytes, namely T helper 1 (TH1) and T helper 2 (TH2) cells, to release cytokines and avoid inflammation. Inflammatory assaults disturb the balance in favor of TH1 against TH2, producing more pro-inflammatory cytokines such as interferon γ, interleukin (IL)-2 and tissue necrosis factor-α (TNF)-α, over the anti-inflammatory mediators such as IL-4, IL-10 and IL-13 (6). This leads to induction of liver production of acute phase reactants such as alpha 1-acid glycoprotein (AAG) and C-reactive protein (CRP) (7, 8). Monocytes are also activated and differentiate into macrophages and dendritic cells, inducing phagocytosis and the further production of cytokines and growth factors. The cascade occurs rapidly and aggressively, lasting for a few days to weeks (7, 8). This process is often resolved once the chemokine gradients are diluted and white blood cells are no longer infiltrating areas of injury, thus initiating the termination sequence, suggestive of rectification of the original insult to homeostasis. These markers and/or changes are typically used as surrogate hallmarks of inflammation and are predictive of treatment outcomes.

Inflammation presents itself in different ways. It can work as a defensive measure in infectious diseases such as sepsis. It also plays a role in the pathophysiology of auto-immune conditions like rheumatoid arthritis (RA) (9). In conditions like osteoarthritis of the knee, where mechanical stress is involved, inflammation is seen as a response to the wear and tear of the synovium and is a driver to the degradation of the joint. Inflammation also occurs in chronic cardiac conditions such as heart failure, and metabolic diseases like diabetes acting as an indicator of worsening prognosis (10, 11). Additionally, inflammatory conditions have been observed in more insidious circumstances such as obstructive sleep apnea, psychological stress, acute mania, and schizophrenia (12–14). In most of these chronic conditions, inflammation is unregulated due to an undefined or repetitive trigger, and acts in a positive feedback loop that perpetuates the disease even further due to a lack of resolution. Under these conditions. inflammation does not resolve on its own, and often becomes uncontrolled, transforming from acute to chronic inflammation.

The molecules which are involved in the inflammatory cascade are hydrophilic signaling messenger proteins known as cytokines, which are released when lymphocytes are activated. They have chemotactic effects, leading to differentiation and communication between immune cells. Pro-inflammatory cytokines such as IL-1β, IL6 and TNF-α mediate inflammation through pattern-recognitions and their respective receptors (15). This causes activation of intracellular signaling pathways which leads to transcription of inflammatory cytokines as well as dysregulation of cell synthesis, and cell death. IL-6 is a primary orchestrator in the initiation of the acute inflammation. It triggers the release of TNF and interleukins as well as acute phase proteins from hepatocytes, which lead to elevated levels of inflammatory signals and biomarkers (16, 17).

Contrary to the harmful consequences of the inflammation-induced reactions, the negative feedback of inflammatory pathways is the production of IL-10. This major anti-inflammatory molecule promotes death of activated inflammatory cells and inhibits synthesis of pro-inflammatory cells such as interferon- γ, IL-2 and TNF α by potentially interfering with their transcription (18). In addition to regulating the inflammatory response, cytokines also influence the expression of many genes including those responsible for hepatic metabolism and drug-receptor proteins.

## Target proteins influenced by inflammation

### Cytochrome P450 enzymes

Cytochrome P450 enzymes (CYPs) are polymorphic proteins bound to a cell and heme component which absorb at the 450 nm wavelengths when exposed to carbon monoxide. They are essential to the biosynthesis of steroids, prostacyclin, and thromboxane A2 (19). CYP enzymes are ubiquitous, however they are mainly expressed in the liver, and small intestine forming CYP1A2, CYP2C9, CYP2C19, CYP2D6, CYP3A4, and CYP3A5 as the predominant forms, which are responsible for metabolizing 90% of drugs (18, 19). Of the different CYP enzymes identified and studied, CYP3A4 and CYP2D6 are considered the most important drug-metabolizing enzymes due to their abundance in the small intestine and the liver as well as their ability to metabolize a plethora of xenobiotic substances (20, 21). Variation in response to therapy can, at least in part, be attributed to differences in the activity and/or the extent of these enzymes (20–22). Drugs and non-drug factors may interact with these enzymes, acting as substrates or leading to induction or inhibition, thus affecting the action and disposition of drugs.

#### Factors affecting CYP expression

Genetic polymorphisms, drugs, dietary components, hormones, and diseases play significant roles in CYP induction and inhibition, thus leading to altered metabolism. Exogeneous factors such as drugs cause the induction of CYP *via* interaction with the pregnane X receptor (PXR), a chemoreceptor, which causes an increase in transcription of CYP DNA and thus an increased production of CYP enzymes (19). For example, rifampin is a potent CYP3A4 inducer, thereby causes decreased serum levels of drugs metabolized by CYP3A4, such as citalopram. Ketoconazole, on the other hand, is a potent CYP inhibitor, leading to an increased and potentially toxic serum concentration of citalopram. This phenomenon has been used in clinical practice to enhance drug therapy. For example, in HIV-1 treatment, the concomitant use of ritonavir leads to the inhibition of CYP3A4, thus when used in combination with a protease inhibitor will increase serum concentrations of the latter (22). The newly approved combination anti-COVID-19 drug, nirmatrelvir plus ritonavir (Paxlovid^®^, Pfizer) presents another example of such drug interactions. Nirmatrelvir blocks the activity of the SARS-CoV-2-3CL protease, an enzyme that the coronavirus uses for its replication. Ritonavir which has no anti-COVID-19 activity, slows down metabolism of nimatrelvir, thus prolongs body exposure to the drug and increases antiviral effectiveness after oral administration (23).

Moreover, dietary intakes, such as grapefruit juice inhibits CYP enzymes in the enterocytes (20). Concomitant use of felodipine, a calcium channel blocker metabolized by CYP3A4, and grapefruit juice leads to a 200% increase in serum concentrations of felodipine. The enzyme alteration is typically reversible within a few days, however, some drug chemicals such as diltiazem and macrolide antibiotics can cause long lasting effects as the CYP enzyme is broken down (20).

In addition to CYP enzyme expression being altered by diet and medications, they are also heavily influenced by genetic polymorphisms, contributing to different drug responses and side effect profiles (24–27). Clopidogrel, for example, is a prodrug heavily reliant on its metabolism by CYP2C19 (24, 25). Different phenotypes of the CYP2C19 gene result in either no enzymatic activity (2 non-functional alleles), intermediate activity (1 functional allele), normal activity (wild type) or increased activity (24). This inter-individual variation causes an unpredictable outcome for clopidogrel and the need for pharmacogenomics to determine optimal dose.

These polymorphisms that lead to altered CYP expression can have grave clinical implications. For example, the United States Food and Drug Administration warns against the use of codeine in individuals classified as rapid metabolizers due to having more than two copies of the CYP2D6 gene (24). This polymorphism can cause increased respiratory depression and death due an increased production of codeine’s active metabolite, morphine (24).

Different CYP activities have been observed in different populations. For example, CYP2C19*3 represents a loss-of-function allele, which is predominantly expressed in some Asian populations. Thus, there is a larger proportion of examined Asians that have intermediate to poor CYP2C19 metabolism (25, 26). This may influence the pharmacological treatment offered to some Asian community.

Sex difference in CYP metabolism has also been observed. In females, CYP3A4 is expressed at a frequency 2-fold higher than in males, which illustrates a 50% increase in the CYP3A-dependent metabolism of drugs such as verapamil (27). CYP2B6, which is responsible for metabolism of many anticancer drugs showed marked interindividual differences in a panel of 80 human liver samples, which revealed higher mean levels of CYP2B6 mRNA, protein, and activity in females compared with males. These sex differences correlated with higher levels of constitutive androstane receptor (CAR) in females, an important regulator of CYP2B6. These findings of sex differences in CYP2B6 protein and activity raise the possibility that CYP2B6 and perhaps also CAR are regulated in a sex-dependent manner by human plasma Growth Hormone (GH) profiles (28, 29).

CYP enzyme expression can also be impacted by the disease severity and type. Conditions that affect the liver, such as non-alcoholic fatty liver disease can cause significant alteration in CYP3A4 expression, and potential changes in the expression of CYP2A6, CYP2B6, CYP2C9, CYP1A2, CYP2D6, and CYP2E (30, 31). In diabetes, a significant reduction in CYP3A4 has been observed, resulting in reduced CYP3A4 dependent clearance (30). Enzymes found in cardiac tissue play a protective role in cardiovascular health *via* the metabolism of arachidonic acid. The expression of the CYP enzymes are also altered in cardiovascular disease. Many chronic conditions such as cardiovascular disease are also accompanied by inflammation which can have further exaggerated effects on CYP enzyme expression (11).

#### Impact of inflammation on CYP expression and Drug clearance

**FIGURE 1:**
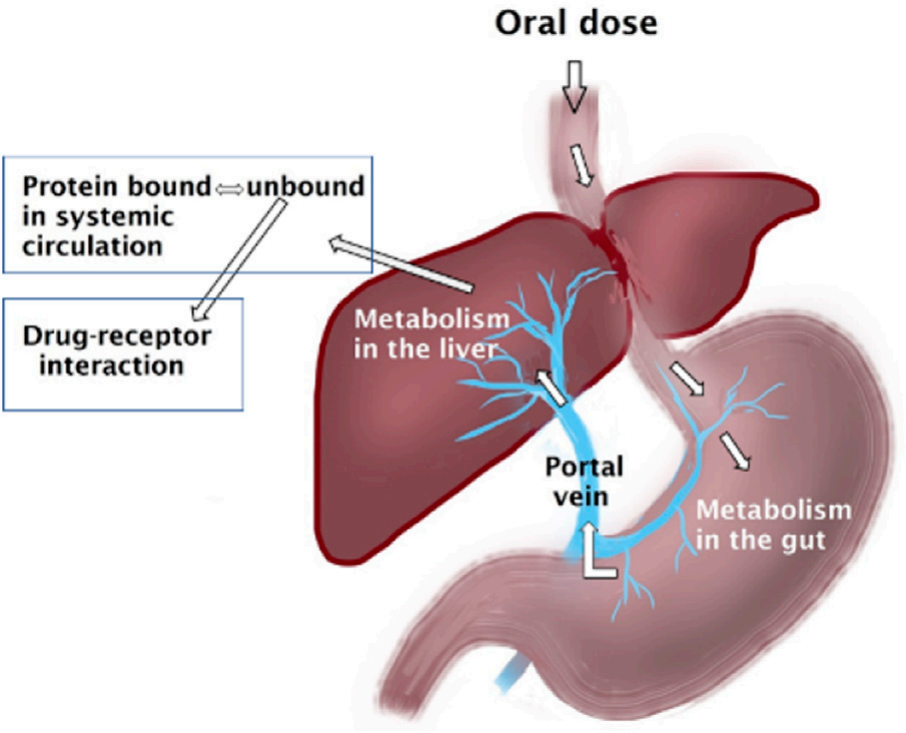
Oral doses of drugs enter the gastrointestinal tract where they can be metabolized. Subsequently the molecules enter the liver *via* the portal vein where further metabolism may take place before entering the systemic circulation where they may get bound to plasma proteins. All these steps can be affected by inflammation.

Drugs are cleared *via* renal and/or non-renal pathways. Following oral doses, depending on the efficiency of the clearing organ, e.g., the gut and liver, drugs may undergo substantial clearance upon the first pass through these organs, substantially affecting their oral bioavailability. Drugs metabolized by the liver are categorized based on the efficiency of their extraction by the liver into drugs with low, intermediate and high extraction ratios. The magnitude of altered drug clearance secondary to inflammation-induced downregulation of CYP and thus reduced hepatic intrinsic clearance is dependent on the extraction ratio of the drug. The clearance of drugs with low hepatic extraction ratio is dependent on the hepatic intrinsic clearance and unbound fraction of the drug, which are both altered by inflammation (Figure 1). On other hand, the clearance of drugs with high extraction ratio are rather dependent on the hepatic blood flow; however, with inflammation, those drugs might become intermediate extraction and their clearance will be dependent on protein binding and intrinsic clearance.

Many studies have reported the effects of inflammation on drug metabolism (32–43). As previously mentioned, inflammation causes increased production of AAG, which when elevated, can bind more circulating basic (cationic) drugs, and reduce the unbound fraction (38). This impacts the distribution and metabolism of basic drugs such as verapamil and β1 blockers, amongst other highly cleared drugs*.* In addition to the effect of acute phase proteins, the cytokine mediated suppression of CYP metabolizing enzymes also affects the circulating concentrations of drugs by lowering the metabolism and allowing drug accumulation and, possibly, toxicity.

With verapamil, higher blood concentrations of the drug have been reported in the rat models such as adjuvant arthritis (AA). In one study, verapamil administered to AA rats demonstrated a 72% higher total area under the plasma drug concentration-time curve (AUC) compared to the control rats (33). The effect of inflammatory mediators on drug AUC is further noted in rats with interferon α2a (IFN)-induced inflammation (36). Rats demonstrated significant rise in TNF-α and NO accompanied with approximately 3.3-fold increased total plasma verapamil concentration. Furthermore, elevated nitric oxide concentrations are associated with higher verapamil AUC (33). This illustrates the relationship between rising cytokine concentrations and higher drug AUC.

A similar relationship exists in humans with Crohn’s Disease (37) and RA (38). In a single centre, single treatment-controlled study, where each patient was given an 80 mg dose of verapamil, the patients with active Crohn’s disease had an AUC increase of 8.8-fold compared to healthy participants and a 5.6-fold increase compared to patients in remission (37). Furthermore, compared with controls, patients with active Crohn’s disease had significantly reduced unbound fraction of both S and R enantiomers. Despite the observed 50% reduction in the fraction of the unbound drug caused by active Crohn’s disease, the free drug concentration remained substantially higher than normal due to the several fold increase in the total drug concentration (37). These active Crohn’s disease patients also had significantly higher CRP and NO levels. Similarly, verapamil concentrations were significantly elevated in patients with active RA compared to healthy controls (38).

Elevated drug concentrations have been observed in experimentally induced inflammatory condition with propranolol, a β-adrenergic blocking agent. Studies found an overall lower hepatic clearance and a great increase in AUC in severe AA rats compared to control rats (39). This effect, like verapamil, has been partially attributed to increased AAG protein binding in an inflammatory state. The progressive reduction in free fraction of propranolol, secondary to elevated AAG, along with the diminished liver capacity apparent in severe arthritis, suggests a culmination of disease severity on drug disposition (39). Therefore, both increased protein binding and decreased intrinsic metabolism must be considered when assessing alterations in the pharmacokinetics of propranolol in severe AA. However, Piquette-Miller et al, found that the addition of ketoprofen improved hepatic metabolic activity and decreased the inflammation-induced changes in propranolol disposition, likely attributed to the downstream effects of non-steroidal anti-inflammatory drugs causing decreased production of inflammatory mediators (39). A similar effect on plasma concentration was seen with acebutolol, which has limited protein binding compared to propranolol (40). There was a three to ten-fold increase in serum concentration of acebutolol observed in rats with AA as compared with controls, which occurred in the absence of significantly increased protein binding suggestive of the predominance of metabolism in the clearance of the drug. This was transient, and levels returned to parallel control concentrations after 2 h (40).

#### Inflammatory mediators involved in altering CYP expression

Many studies have shown that elevated levels of pro-inflammatory cytokines such as TNF-α, IL-6, influence the transcription of CYP enzymes (41). Although the exact mechanism is not fully understood, theories of the roles of pro-inflammatory mediators on drug metabolizing enzymes mRNA have been explored. In 1994, Shedlofky et al who studied the effect of pro-inflammatory Gram-negative endotoxin (LPS) injections on human volunteers, found the depression of drug metabolism is developed over a 24 h period. The inflammatory response and the extent of the depression correlated with the intensity of the inflammatory response as illustrated by elevations of TNF-α and IL-6 as well as decreased clearance of substrate drugs (42). In addition, animal studies have noted that cytokine activation of hepatic Toll-Like receptor 4, cause suppression of drug metabolizing enzymes including CYP3A1 (43). It appears that inflammation also downregulates PXR, a nuclear receptor associated with gene expression involved in drug metabolism (44). Once activated by a ligand (such as drugs, herbals dietary supplements), PXR heterodimerizes with retinoid X receptor (RXR) to form a transcriptional complex that affects the expression of genes of certain CYP3A enzymes, and multiple hepatic transporters required to metabolize and clear xenobiotics (44–46). This receptor can be directly downregulated or be affected by the suppression of RXR during inflammation *via* cytokines, directly affecting the transcriptional activity of CYP enzymes. PXR can also affect and be manipulated by other transcriptional factors seen in inflammation such as Nuclear Factor Kappa B (NF-KB) which is involved in the production of proinflammatory mediators (45).

NF-KB is a regulatory transcription factor in inflammation which can suppress or be suppressed by the activity of PXR and has been also associated with interference of CYP expression (CYP2C11, CYP2E1) (47). NF-KB is activated during inflammation through the activity of circulating cytokines released by immune cells responding to an insult. It directly disrupts the RXR complexation to DNA sequences, thus suppressing CYP3A4 expression (48).

TNF-α is also believed to play a role in influencing transcription factors and proto-oncogenes associated with CYP production (49). TNF-α is involved in initiating the inflammatory cascade by inducing the production of other cytokines such as IL-1 and IL-6, and is involved in the migration of monocytes into affected tissue, and proliferation of T lymphocytes (50). It is a major player in hepatic inflammatory disease such as alcoholic hepatitis (51). In terms of CYP expression, an inverse correlation between TNF-α and CYP2C19 activity has been observed in heart failure patients (52). In HepaRG cell studies, it is noted that TNF-α exposure does not cause a significant immediate change in CYP enzymes, but requires 24 h for profound effects (53).

**FIGURE 2:**
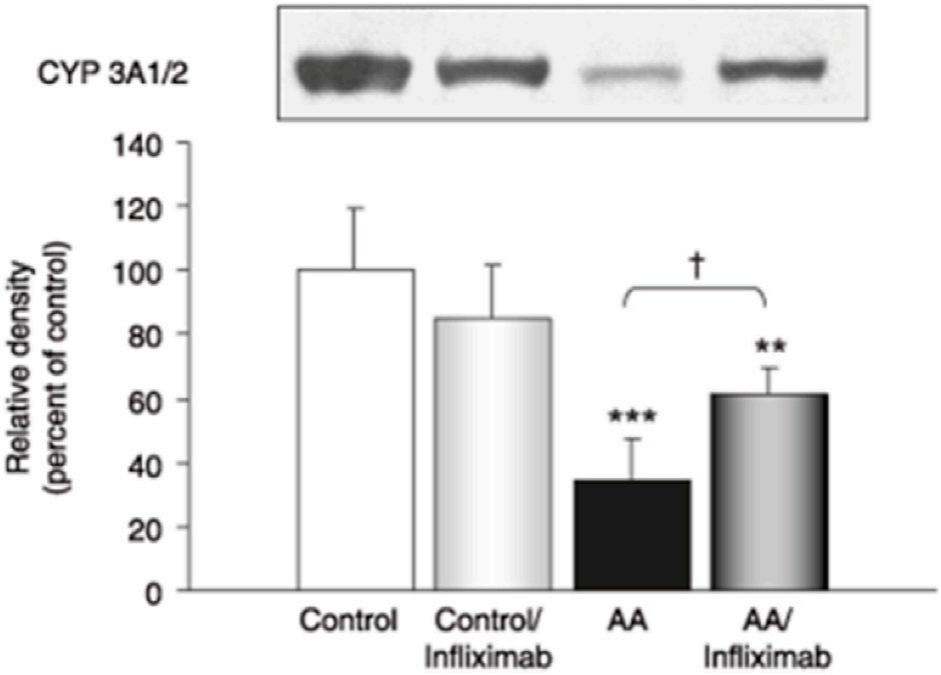
Western blot and relative density of CYP 3A1/2 enzymes in AA rats treated with and without infliximab. Adapted from (35).

Moreover, the administration of anti-TNF-α agents such as infliximab in rat models with induced arthritis has shown a partial restoration of CYP3A and CYP1A activity previously suppressed by the inflammatory state, as illustrated by an increase in CYP concentration and density (Figure 2) (35). Rat models have shown that soluble TNF-α regulates CYP3A by interacting with TNF-α receptor 1, which activates NF-KB, leading to potential transcriptional changes to promote inflammation and cause CYP modulation (54, 55).

TNF-α also impacts the release of other inflammatory mediators that affect CYP expression such as the production of NO mediated synergistically with other cytokines (8). NO also plays a key role in the pathogenesis of inflammation. Under normal conditions, NO elicits anti-inflammatory effects, however, once overproduced *via* cytokines, it acts as a pro-inflammatory mediator, facilitating hyperinflammation (56). Studies have shown reduced metabolism of drugs due to NO’s effects on CYP enzymes. In *in vivo* studies, bacterial lipopolysaccharide (LPS) was used to increase NO synthesis, which was correlated with decreased hepatic CYP activity (57). This was confirmed *via in vitro* studies treating hepatic microsomes with NO, leading to a suppression of CYP reactions *via* interaction with Fe^2+^ and Fe^3+^ hemes in the enzymes (57). Excess NO production has also been shown to be triggered by IL-1β and its interaction with inducible NO synthase in rat cardiomyocytes (58, 59). IL-1β is vital for lymphocyte activation, and is typically required for T-lymphocyte proliferation, along with enhancing the cytokine production by the T lymphocytes (60). It is responsible for producing fever, and initiating the production of acute phase proteins from hepatocytes (60). IL-1β induces NO synthase, which affects CYP expression, but can also suppress CYP enzymes such as CYP2C11 independently of NO activity in rat hepatocytes (61). Furthermore, cynomolgus macaque hepatocytes, which have similar CYP enzyme expression as human hepatocytes, had significantly reduced expression of CYP1A1, CYP2C8, and CYP2C19 mRNA when treated with IL-1β (62). Similar results were found in human hepatocytes, except CYP1A2 response was variable activity amongst the different donor tissues (63).

Furthermore, the enhanced production of cytokines by IL-1β in T lymphocytes leads to the production and release of IL-2, which is involved in augmenting B-cell proliferation and antibody production (61). In terms of CYP expression IL-2 seems to have a transient suppressive effect on CYP3A enzymes that is overcome after 24 h (64). However, IL-2 also has an indirect effect on CYP expression by increasing levels of IL-1 and IL-6, which contributes to the suppression of CYP enzymes. In cryopreserved human hepatocytes, IL-2 had minimal effect on CYP isoforms, apart from CYP2D6 mRNA, which was elevated (65).

IL-6 is considered a primary cytokine in hepatic response to inflammation as well as a regulator of acute phase protein biosynthesis in hepatocytes (49). Recombinant IL-6 has been shown to reduce CYP activity as well as block CYP2B1 and CYP2B2 mRNA induction in rats (66, 67). Additionally, Klein et al. demonstrated that CYP1A2 expression was decreased by 75%, CYP3A4 by 80%, CYP2C9 by 60%, and CYP2D6 by 50% after 24 h in HepaGR cells treated with IL-6 (53). CYP2E1 was increased by 402% in PHH cells, but this was not reflected in the HepaGR line. In addition to the decreased mRNA expression, decreased CYP activity was elucidated by quantification of the formation of drug metabolites. The CYP isoform activities had variable response to IL-6 challenge, for example, CYP2B6 significantly decreased formation of OH-Bupropion at 48 and 72 h where CYP2C8 had a less pronounced effect (53). Moreover, the effects of the IL-6 challenge continued to a greater extent after 72 h. The model in this study used IL-6 at 10 ng/mL for maximum effect, which is higher than what is found in inflammatory conditions, (e.g., 0.5 ng/mL in rheumatoid arthritis synovial fluid) (68). Additionally, IL-6 shares the receptor subunit, gp130, with other proinflammatory cytokines. Once bound, this subunit is responsible for the initiation of transduction cascades that lead to the upregulation of acute phase proteins such as CRP (49). Furthermore, IL-6, markedly suppressed mRNA levels of CYP1A1/2/3 in human cell lines (66).

CRP is involved in upregulation of adhesion molecules, monocyte recruitment, and complement activation in inflammatory disorders (66). In addition to immunomodulation, CRP is also used as a marker for disease prognosis in conditions such as post-myocardial infarction management, where patients with higher levels of CRP at discharge are more likely to have a history of unstable angina and have symptom onset at lower levels of activity. Moreover, it has been correlated with altered drug metabolism in patients with hemodialysis (69). According to recent evidence, CRP modulates CYP activity by upregulating multiple microRNAs (miRNA) in hepatic tissue during inflammation which act as negative gene regulators, causing post transcriptional suppression *via* mRNA degradation or translational inhibition, and in turn suppresses CYP genes expression and activity (70, 71). In Table 1 the effect of inflammatory mediators on mRNA expression of predominant CYP isoforms is summarized.

**TABLE 1:** Examples of the effect of inflammatory mediators on mRNA expression of predominant CYP isoforms.

Inflammatory mediator	Model	CYP isoform mRNA expression	References
TNF-α	TNF-α challenge of human hepatoma cell line (FLC-4)	CYP3A4 (↓51%)	(72)
IL-6	IL-6 challenged human—HepaRG cells	CYP1A2 (↓90%), CYP2C9 (↓83%), CYP2D6 (↓50%), CYP2C19 (↓83%), CYP3A4 (↓93%)	(53)
IL-2	IL-2 challenged in human hepatocytes	CYP3A4 (↓70–90%)	(64)
IL-2	IL-2 infusion in patients with hepatic metastasis	CYP1A2 (↓63%), CYP2C (↓55%), CYP2E1 (↓40%), CYP3A (↓61%)	(73)
IL-1β	IL-1 β challenge in human hepatocytes cell culture	CYP1A2 (NS), CYP2B6 (NS), CYP2C9 (NS), CYP3A4 (↓95%)	(63)
IL-1β	IL-1 β challenged in human—HepaRG cells	CYP3A4 (↓97%), CYP1A2 (↓93%), CYP2C9 (↓90%), CYP2C19 (↓93%), CYP2E1 (↓73%)	(52)

NS, no significant change.

### Impact of inflammation on drug transporters

**FIGURE 3:**
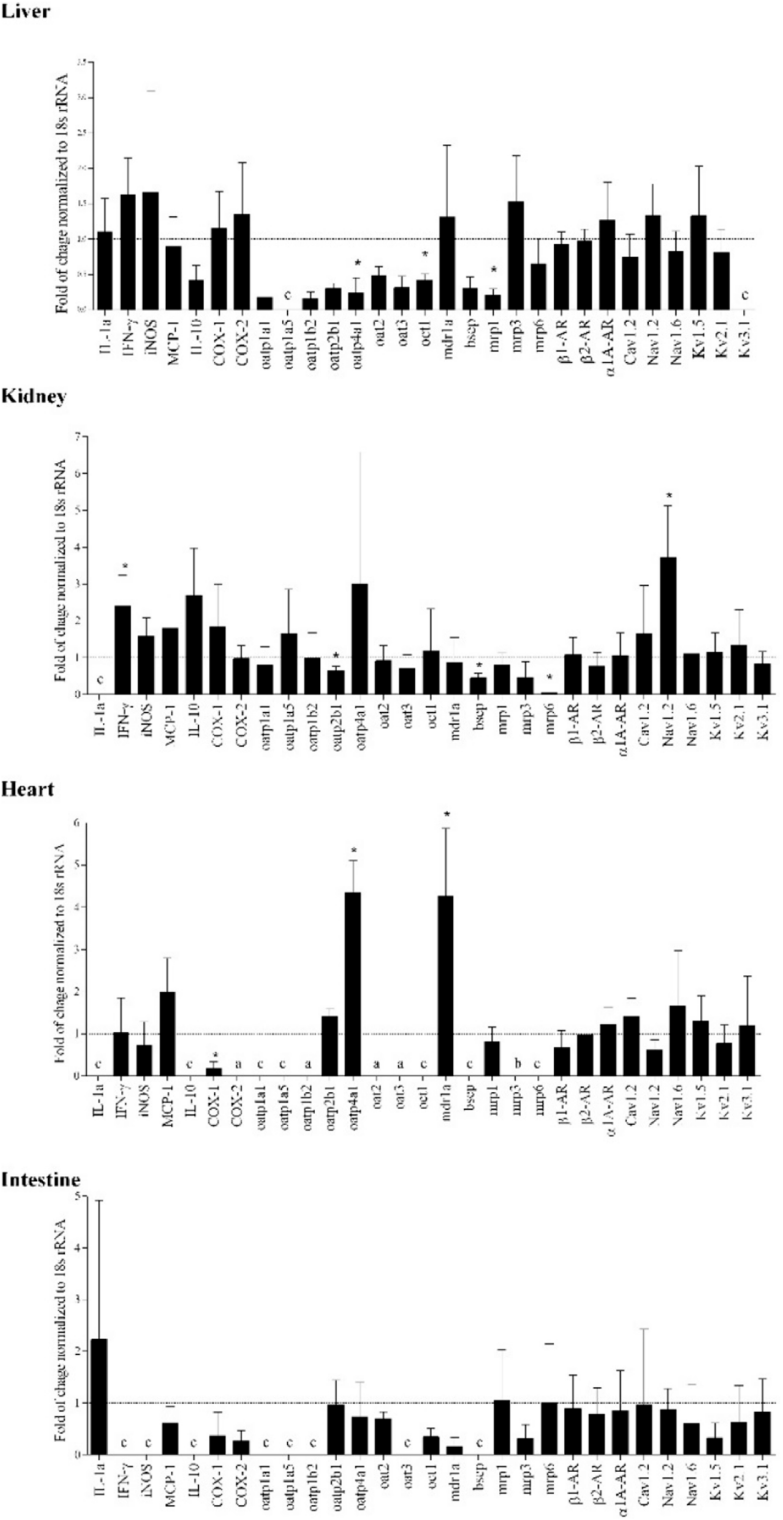
Adjuvant arthritis influences the tested molecular targets and transporters’ gene expression in various rat organs as determined using real time polymerase chain reaction (RT-PCR). Gene expression changes are represented by fold of changes of target genes in adjuvant arthritis rats compared to Control (dotted line) (*n* = 4/group). Gene expression was normalized to 18s rRNA. *, *p* < 0.05 vs. control rats. a, detectable in AA only; b, detectable in Control group only; c, absent in both healthy and AA. From (74).

Inflammation is also associated with altered transporter gene expression in addition to CYP expression (Figure 3) (74). Drug transporters play a vital role in the absorption, distribution, and elimination of medications. Transporters are a part of two superfamilies; solute carrier (SLC) transporters and ATP-binding cassette (ABC) transporters, found across various tissues which coordinate and regulate the flow of molecules across membranes (75). In terms of function, the SLC transporters typically are involved in the influx of molecules into cells and tissue, whereas ABC transporters are commonly efflux transporters, with both families containing multiple subtypes. During inflammation, evidence suggests these transporters are downregulated in the liver and kidney, specifically, there is downregulation of influx transporter genes organic cation transporter (OCT)-1, organic anion-transporting polypeptide (OATP)4A as well as efflux transporter gene, MRP1, in the liver. This suggests a change in extraction and intrinsic clearance of the drug as less is passing through the liver. The change in extraction ratio was seen experimentally with propranolol and verapamil, changing from high and intermediate to low extraction drugs, respectively (35, 39). Additionally, cellular models have shown that transporters such MRP2 in the jejunum were also downregulated in general inflammation, with other transporters exhibiting time-dependent suppression, such as ABCB1 which is only downregulated for the first 48 h (76).

In chronic inflammatory conditions such as RA, human cell studies have observed downregulation of transporters such as OAT1B1, with a marked reduction in ABCC2 by 50%, and SLC10A1 by 75% compared to control as a result of IL-6, which is typically elevated in RA (77, 78). This in turn results in lower uptake of medications like fluvastatin for hepatic clearance, leading to a total decrease in clearance of the drug by 40%–50% in RA patients (77). However, the plasma concentrations of CRP or cytokines does not correlate with the pharmacokinetic parameters of fluvastatin when the analysis was performed. Further, there is no significant differences in the unbound fraction that is mentioned. In other inflammatory conditions such as ulcerative colitis (UC) human biopsy studies have found that there was a significant 3-fold reduction in ABCB1 efflux transporters, along with a 6-fold decrease of ABCG2 (76). These transporters are regularly involved in the efflux of UC medications, and due to their downregulation, there is elevated concentrations of drugs used in UC such as steroids, 5-aminosalicylate, and cyclosporine A. This sentiment has been echoed in other animal-based studies where other efflux transporters found in the liver and intestines, such as P-gp, were downregulated by approximately 50% in UC (79). As a result of the downregulation, when the UC rats were administered cyclosporine A, there was an approximately 1.78-fold increase in plasma drug concentration compared to control.

Furthermore, exposure to 100 ng/mL TNF-α or 10 ng/mL IL-6 for 48 h was found to down-regulate mRNA levels of major sinusoidal influx transporters, including sodium-taurocholate cotransporting polypeptide, OATP1B1, OATP1B3, OATP2B1, OCT-1, and organic anion transporter 2 (80).

### Impact of inflammation on drug-receptor target proteins

Despite much work done on the effect of various factors on the CYPs formation and clearance, drug-receptor target proteins have been largely ignored in this context.

There is no reason for proteins other than CYPs to remain unaffected by inflammation. Indeed, down-regulations of various cardiovascular target proteins have been reported (Table 2). Such down regulations are bound to have clinical consequences with respect to pharmacotherapy. For example, the cardiovascular effect of verapamil is shown to be reduced in humans with active arthritis (38), obesity (81) and in old age (82). Surprisingly, in all these studies the reduced effect of verapamil has been observed despite the elevated drug concentration. The increased drug concentration is expected due to the down regulation of CYPs needed to clear the drug as well as increased drug plasma protein binding. Later data generated from experimental animals demonstrated that these lower than expected effects were due to downregulation of calcium channel target proteins that are essential for the effectiveness of verapamil (36). These observations suggest that inflammation down regulates both of the involved proteins, the one responsible for its clearance, as well as the one needed for the drug-receptor interaction.

**TABLE 2:** Effect of inflammation on the pharmacodynamics and pharmacokinetics of select drugs.

Drug	Effect of inflammation on			Species/tissue/cells
	Clearance	Target protein	Therapeutic outcome	
Verapamil	Decreased	Down-regulation of L-type Calcium Channels	Reduced effect	Humans and rat (36–38, 82–84, 87)
Propranolol	Decreased	Down-regulation of β1 and β2 receptor	Reduced effect	Rat (32)
Bupropion	Unknown	Unknown effect on noradrenaline and dopamine reuptake inhibition	Unknown	HepaRG cells (52)
Tramadol	Decreased active metabolite	Unknown effect on µ receptor	IL6-dependent Reduced drug tolerance	Human (88)
Sotalol	No change	Down-regulation of β1 and β2 receptors	Reduced effect	Rat (85)
Nebivolol	No change	No effect on β3 receptors	No effect	Rat (86)
Losartan	Lower concentration of active metabolite	No effect on AT_1_R receptors	No effect	Human (107)
Valsartan	No change	No effect on AT_1_R receptors	No effect	Human (109)

The receptor downregulation is also observed for propranolol administered to rats with adjuvant arthritis (32). Furthermore, sotalol, which has low plasma protein binding and is renally eliminated, also demonstrated a reduced PR interval in AA rats compared to control, suggestive of downregulation of the drug’s target receptor in the absence of protein binding involvement (85). This effect on the pharmacodynamics, however, was reversed when inflammation was controlled with the administration of infliximab (35). Verapamil’s reduced effect in extending PR intervals in AA rats was also reversed when combined with valsartan, an antagonist to the proinflammatory mediator; angiotensin 2 (83).

For the downregulation of β1-adrenergic receptors cause by inflammation (32), a link with norepinephrine transporters is suggested (89). Hyperactivity of sympathetic nervous system has been shown in rheumatoid arthritis with a potential link to the cardiovascular mortality (90–92). In addition, the norepinephrine transporter functionality has a strong role in dictating the distribution of sympathetic innervation (93).

**FIGURE 4:**
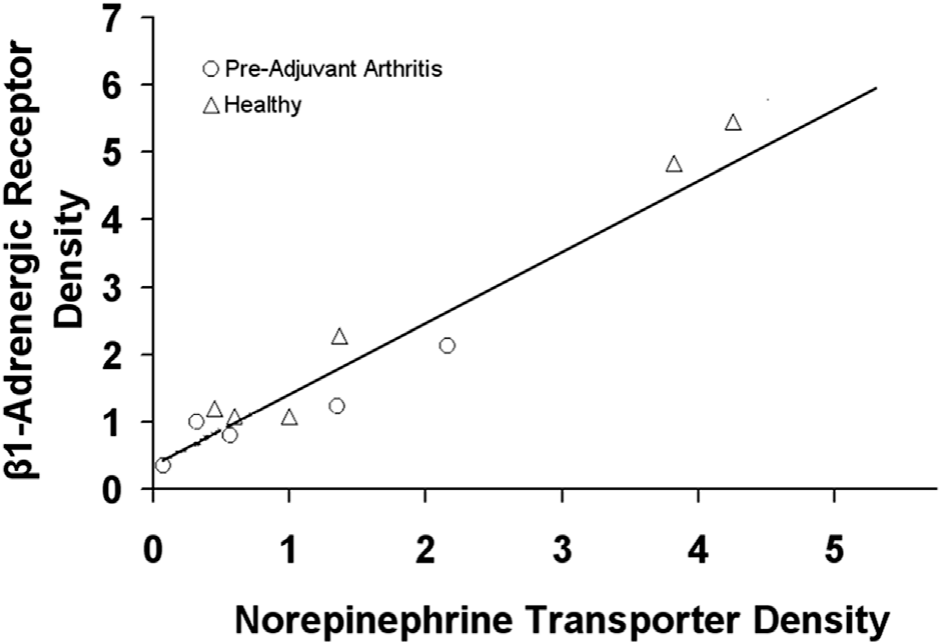
The relationship between the cardiac β1-adrenergic receptor and norepinephrine transporter density in healthy rats and rats with experimental arthritis (Pre-Adjuvant) rats. Adopted from (89).

It has been shown that, in experimental inflammation, not only are the cardiac β1-adrenergic receptors target proteins down-regulated, but the norepinephrine transporters are also reduced (Figure 4) (89). Such a down regulation in the neurotransmitter receptor is proposed to cause a reduced uptake of norepinephrine by the nervous system.

## Pharmacotherapy outcome of inflammation

Studies of altered CYP expression due to inflammation has led to generation of data related to certain inflammatory disease states as well as special populations. Of note are conditions such as RA, Crohn’s disease, ulcerative colitis, cancer, obesity, diabetes, HIV, acute infections, COVID-19 and renal insufficiency. Additionally, these conditions are often associated with severe chronic pain. As the dysfunction in balance between the pro- and anti-inflammatory mediators is commonly seen in chronic pain, there may be further exacerbation of the inflammatory conditions and their effects on the drug action and disposition that is unrecognized (2). Altered inflammatory mediators balance (2) may also be involved in the delayed pain-induced gastric emptying which results in late onset of action of analgesics (99).

### Rheumatoid arthritis (RA)

**FIGURE 5:**
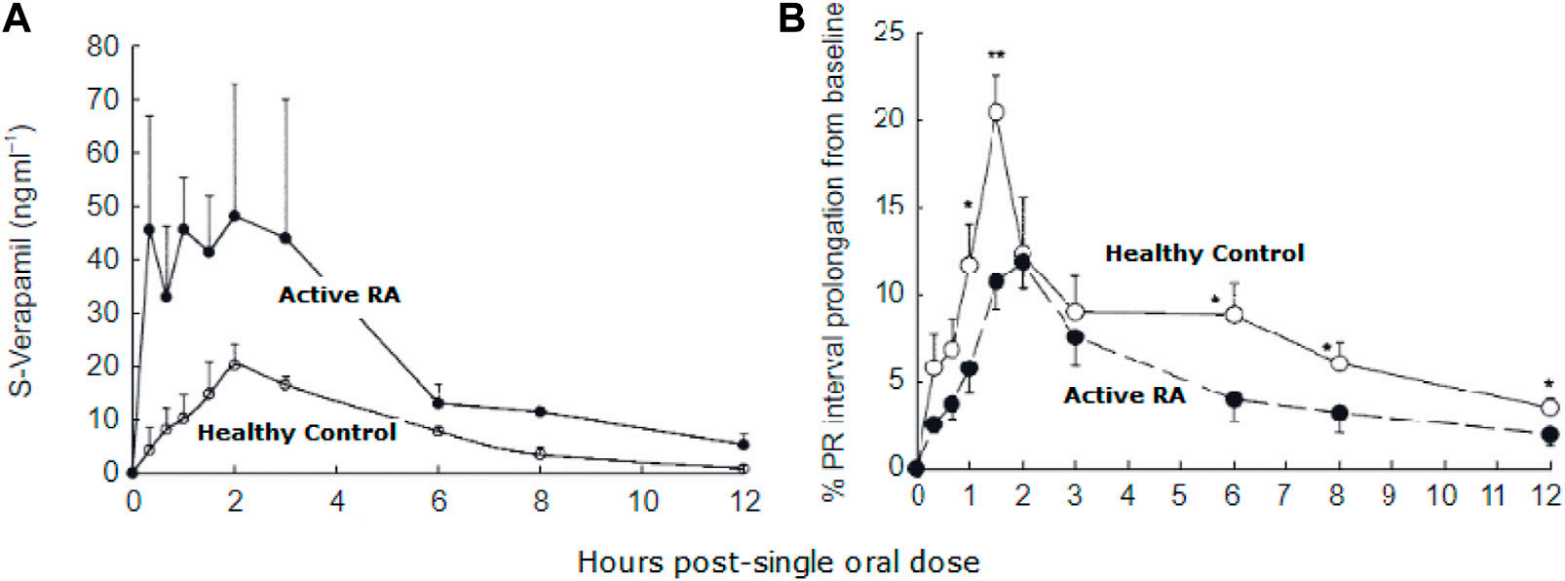
Plasma concentration of S-verapamil in patients with active rheumatoid arthritis (RA) and healthy controls **(A)**. Despite increased plasma concentration, the pharmacological effect of verapamil was reduced in RA patients compared to healthy controls **(B)**. From (37).

Given that a significant proportion of patients with RA also experience cardiovascular diseases (94, 100, 102), more attention should be paid to the effect of inflammation on the pharmacokinetics and pharmacodynamics of drugs to avoid treatment failure and poor prognosis. Patients suffering from active RA have been found to exhibit altered pharmacokinetics of different cardiovascular medications. It has been reported, as compared with healthy subjects, they have higher concentrations of verapamil due to decreased clearance, but interestingly, show reduced dromotropic effect which is suggested to be due to down-regulation of the target receptor proteins (37) (Figure 5).

These effects are normalized when the disease and/or inflammation is brought under control due to remission and/or pharmacological intervention. Ling et al. have found no significant difference in PR prolongation in response to verapamil between healthy subjects and patients who have their RA controlled by using infliximab or conventional anti-inflammatory drugs (84). A PR interval prolongation indicate blockade of the calcium channels. Data generated from experimental arthritis confirm normalization of CYP1A and CYP3A isoenzymes down regulation by infliximab (35).

**FIGURE 6:**
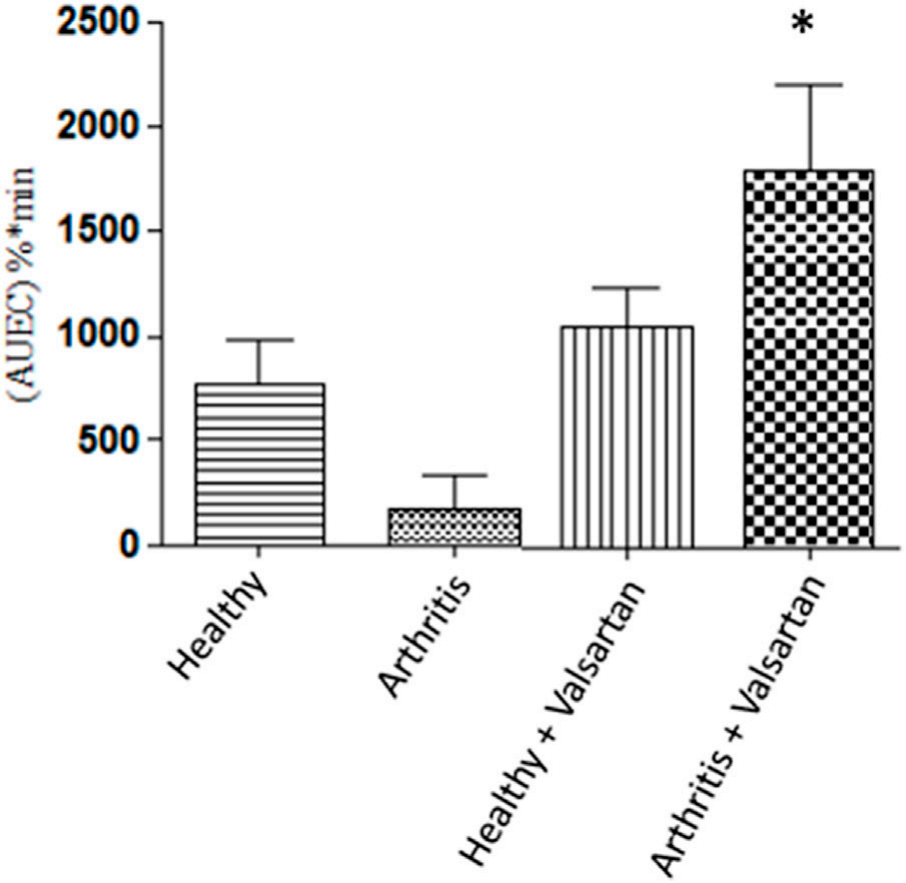
The effect of valsartan on the P-R prolongation activity of verapamil. Healthy and adjuvant arthritics rats were pre-treated with valsartan administered verapamil and the P-R interval area under effect-time curve measured. The effect on the arthritic rat treated with valsartan was significantly greater than both untreated healthy and arthritic rats. From (83).

Experimental animal studies have demonstrated that the inflammation-induced down regulation of calcium channels receptors is reversed by angiotensin II receptor antagonist valsartan [Figure 6, (83)], likely through the drug’s direct free radical scavenging and indirect anti-inflammatory actions by inhibition of the pro-inflammatory mediator angiotensin II (109). This observation is in line with the clinical practice of combination therapy (110).

Fluvastatin is an HMG-CoA reductase inhibitor (statin), which exhibits higher plasma concentrations in RA than in healthy subjects. This has been shown to be due to inhibition of OATP1B1 transporter which is inhibited by the disease (77). Furthermore, higher exposure for another statin, simvastatin, in RA is attributed to inhibition of its metabolism that appear to normalize by the anti-inflammatory sarilumab (78). Whether the increased statins concentration in RA causes increases in the incidence of myalgias remains to be seen.

It is well acknowledged that a major contributor to the CYP suppression in RA is linked to the elevated levels of IL-6. This is also further supported by the administration of biologics designed to target IL-6 or IL-6 receptors, such as tocilizumab, ruxolitinib, or sarilumab, resulting in restored CYP function along with lower drug plasma concentrations compared to untreated RA patients (101–106).

Data generated using experimental arthritis suggest that the disease affect β1 antagonists such as propranolol in a fashion similar to the verapamil observation, i.e., increased concentration but reduced effect (Table 2).

It is important to note that the effect of angiotensin interrupting drugs such as losartan (107) and valsartan (108) is not altered by arthritis suggestive of lack of influence of the disease (i.e., inflammation) on the related receptor (Table 2). Another equally interesting finding is regarding nebivolol, a third generation β-adrenoceptor blocker with high selectivity for blocking β1 and β3-agonistic properties. In experimental arthritis, neither action nor disposition of nebivolol is reported to be influenced by inflammation despite the reduced β1-AR expression, but no change in that of β2 and β3-AR (86). This is suggestive of the predominance of contribution of β2 and β3-AR to the action of nebivolol on blood pressure. In addition, the lack of an inhibitory effect of inflammation on the clearance of nebivolol is suggestive of mechanisms other than an efficient hepatic metabolism for its low bioavailability. Thus, like the AT1 antagonist, e.g., losartan and valsartan, a β2 and β3-AR blocker is a suitable choice to control blood pressure in the presence of inflammatory condition.

RA patients experience cardiovascular complications substantially higher than the general population (94), and the receptor proteins alteration is only a part of the effect of inflammation on the cardiovascular system. For example, inflammation is reported to influence two other important pathways involved in regulating cardiovascular function, namely the arachidonic acid metabolism and the renin angiotensin system (RAS). Inflammation brought about by adjuvant arthritis is shown to cause tissue-dependent imbalances of arachidonic acid metabolites. It elevates the ratio of cardiotoxic 20-hydroxyeicosatetraenoic acid over cardioprotective epoxyeicosatrienoic acids in the heart but reduces the ratio in the kidney (95).

**FIGURE 7:**
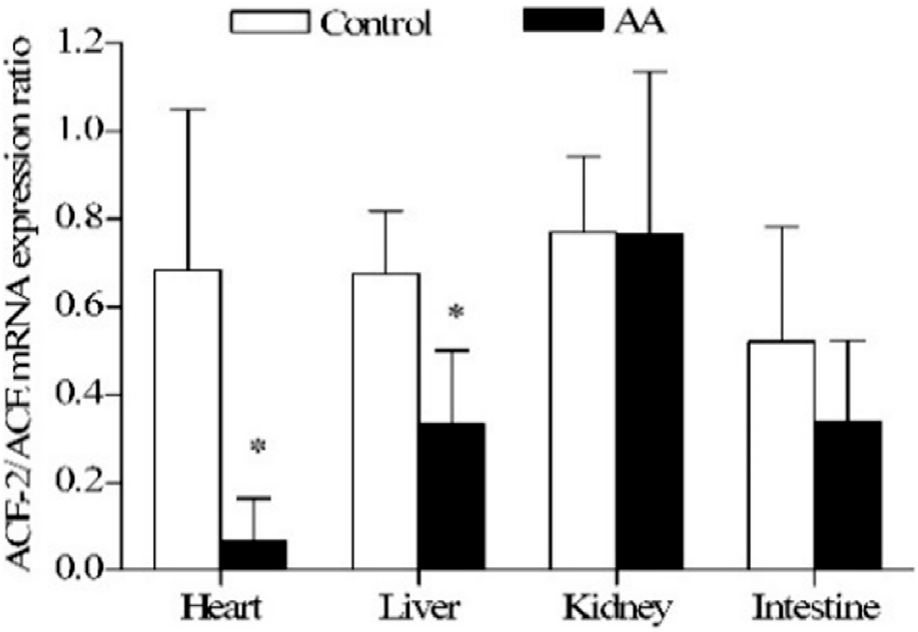
Effect of adjuvant arthritis on ACE-2/ACE constitutive gene expression ratio in different rat organs. *, *p* < 0.05 vs. control rats. AA, adjuvant arthritis. From (97).

The hormonal system RAS controls cardiovascular function by maintaining balances in body fluid volume, blood pressure in both health and disease (96). Inflammation causes imbalances in the gene expression of angiotensin converting enzymes toward cardiotoxicity (97) (Figure 7). In addition, the peptides involved in the RAS are also affected. The cardiac and renal biologically active peptides (Ang II and Ang1-7) and the target proteins involved in the peptide-receptor binding (Ang II type 1 and type 2, and Ang1-7 receptor, Mas) are altered toward cardio-renal toxicity (98).

### Crohn’s disease and ulcerative colitis

Crohn’s disease is an inflammatory bowel condition which involves an intestinal insult that disrupts barrier function in the gastrointestinal tract, thus affecting absorption and potentially drug metabolism (37). It is associated with many cardiovascular manifestations, such as heart failure, pericarditis, thromboembolism, which are typically treated with CYP substrates (113). Given that the intestine is involved in first pass metabolism, and highly concentrated with CYP3A4, the effect of hyperinflammation may influence the treatment efficacy (111). In one study, the authors found reduced CYP3A4 content in the intestinal biopsies from patients with active Crohn’s disease (112). Ulcerative colitis (UC) is a condition similar to Crohn’s disease but is usually restricted to the large intestine. Its symptoms include erosion and ulcers in the intestinal mucosa (76). The chronic inflammatory state in the gastrointestinal tract of animal and UC patient causes a significant reduction in CYP3A, CYP2C9, UGT1A, and drug transporters genes such as ABCBG2, ABCB1, and SLC16A1 which downregulate transporters such as P-GP (76,112–114). These enzymes are important for the bioavailability of UC medications, as many of them are substrates for those transporters, and could be attributed to the altered drug concentrations in UC patients. The therapeutic consequences of these finding remain to be studied.

**FIGURE 8:**
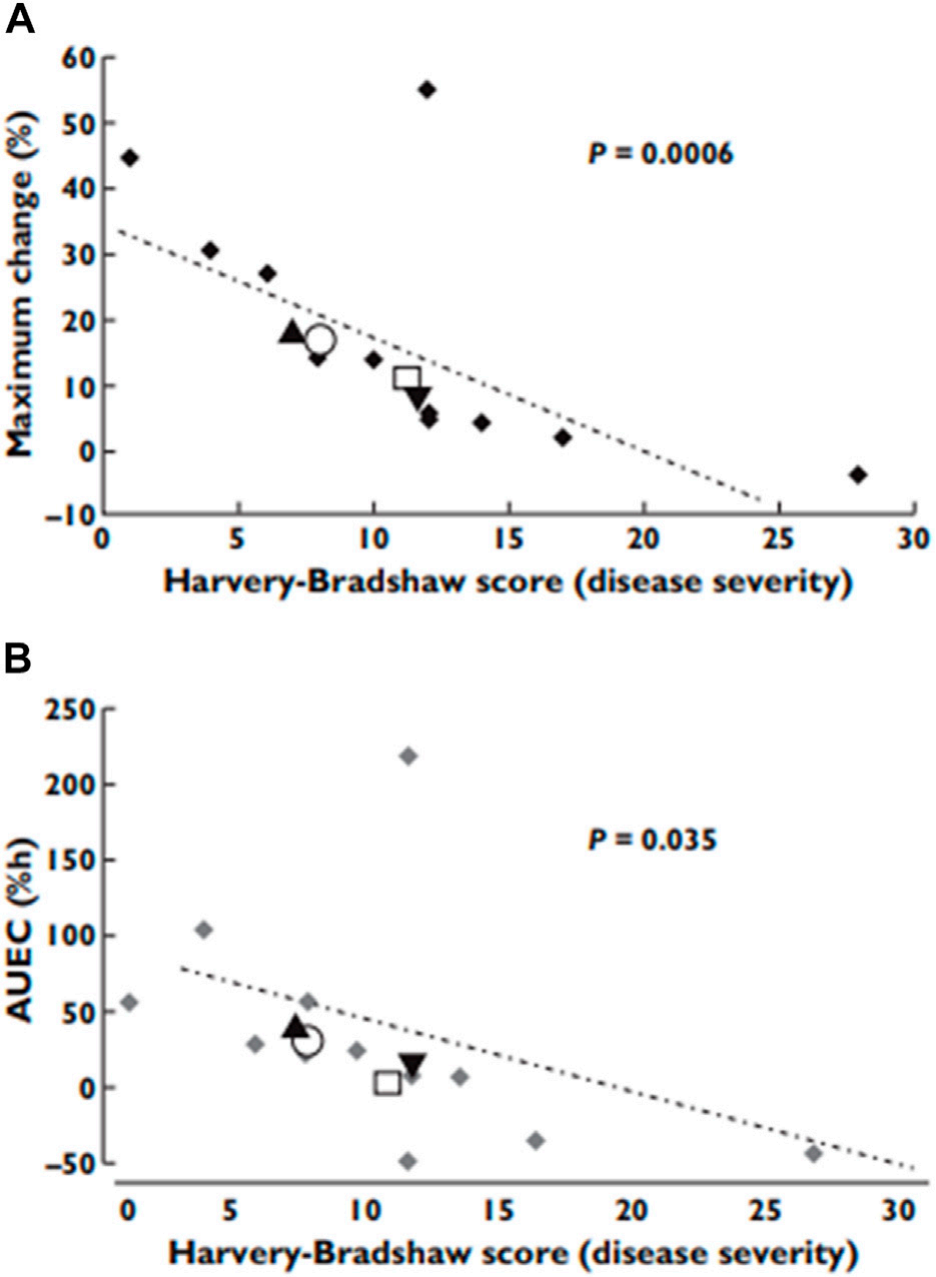
The effect of Crohn’s disease severity on verapamil pharmacological effects. **(A)** the observed maximum effects; **(B)** the area under effect−time curves. From (37).

Another report reveals that Crohn’s disease caused elevation of verapamil concentration in plasma. However, indeed, the efficacy of verapamil to prolong PR interval was very much dependent on the disease severity, the higher the severity the less the efficacy (Figure 8) (37). This observation is in line with what has been reported for other inflammatory conditions, e.g., RA. Once the disease (i.e., inflammation) is controlled, the downregulating effect on the calcium channels is reduced.

### Cancer

Cancer, is also associated with inflammation as tumor cells release cytokines and chemokines which recruit leukocytes and other proinflammatory mediators (115). Also, CYP enzymes are involved in both the pathogenesis and treatment of cancer, as they metabolize carcinogens as well as activate anticancer drugs.

The effect of inflammation on the pharmacokinetics of anticancer drugs is relevant when considering treatment options for cancer patients as many antineoplastic agents have a narrow therapeutic window and are either metabolized or activated by CYPs. For instance, the chemotherapy agent docetaxel is predominantly cleared by CYP3A4. Severe toxicity has been shown to be 3-fold higher for patients with elevated inflammatory markers (AAG and CRP) (116). It is postulated that the higher the levels of AAG, the greater the inflammatory response and thus, the more suppression of CYP metabolism hence greater toxicity (117). The suppression of CYP3A4, has been observed in the presence of specific elevated cytokines such as IL-6, TNF-α and CRP (118). Mediators such as IL-6 and TNF-α can act as biomarkers for impaired clearance as elevated in cancer patients (119). Mimura et al. have reported that the treatment of three-dimensionally cultured functional liver cell line with IL-1b or IL-6 caused a concentration-dependent decrease in CYP3A4 protein expression and its catalytic activity (72). TNF-α treatment had no effect on CYP3A4 protein expression or its catalytic activity (as measured by formation of triazolam metabolite, hydroxy-triazolam) although it did decrease mRNA. The cell model illustrated modulation of CYP3A4 expression by cytokines, increased toxicity of chemotherapy agents by IL-6, and restoration of CYP3A4 mRNA expression by anti-cytokine agents (72).

It appears that malignant tissues have increased expression of CYP enzymes such as CYP2E1 and CYP3A4 (120–123). Cancers such as those of advanced ovarian breast and coflorectal cancers are associated with upregulation of certain CYP enzymes. CYP2E1 activity measured by 6-hydroxychlorzoxazone/chlorzoxazone ratio has been reported to be markedly up-regulated in cancer (1.30 vs. 2.75). This is while CYP3A phenotypic activity measured by omeprazole sulfone/omeprazole ratio is shown to be reduced in cancer (0.23 vs. 0.49) (122).

The therapeutic outcome of these changes in CYPs, and other important target proteins is mainly unknown although their possible involvement in the variation in response cannot be ruled out. Moreover, more recently, targeted immunomodulators that are being used to make treatment more precise, enhance cytokine release (131), thus potentially influencing efficacy of other drugs.

### Obesity

Obesity is an independent risk factor for many diseases, including hypertension, diabetes, arteriosclerosis, arthritis, among others. Adipose is an active endocrine and paracrine tissue which releases adipokines, pro-inflammatory cytokines as well as NO (124). Increased plasma CRP concentrations and elevated inflammatory markers have been illustrated in obese patients, resulting in CYP downregulation (125). In addition to the changes in metabolizing enzymes, obesity is often associated with hyperlipidemia, leading to a higher concentration of lipoproteins. Given that many drugs have a high binding capacity to lipoproteins, an excess of it may be associated with a higher total drug concentration. However, there is a paucity of studies looking at lipophilic drugs to assess this possibility. In addition, a very few studies have focused on the therapeutic relevance of these obesity-driven changes (81, 130).

Rise and fall in TNF-α and IL-6 have been found to be correlated with weight loss, respectively (126). Both these cytokines are linked to insulin resistance, endothelial dysfunction, and cardiovascular disease, thus involved in the pathophysiology of diabetes and hypertension (124). Animal studies have shown an isozyme specific effect on CYP enzymes with obesity, with a decrease in CYP3A, and increase in CYP2E (127–129). Additionally, some mouse studies have also illustrated a downregulation of CYP1A1, and steroidogenic acute regulatory protein in obese mice, resulting in altered steroid metabolism and deficiency in the mouse’s sperm (127).

Rat models on a high fat diet have shown reduced concentrations of a lidocaine metabolite consequent to reduced expression of CYP3A1, CYP1A2, CYP2C12 (131). Moreover, rats on high fat diets for 40 weeks showed a 43% decrease in CYP enzyme expression (130). A study on obese human subjects, on the other hand, attributes the changes in the pharmacokinetics of lidocaine to increased volume of distribution consequence of increased body volume in obesity (131).

Evidence for altered pharmacokinetics of drugs in obesity of humans and experimental animals is abundant (e.g., ibuprofen, salicylates, brexiprazole, morphine, amoxicillin, fluconazole, triazolam) (125). Unfortunately, however, due to the lack of corresponding pharmacodynamic data, the clinical outcomes of these finding have remained mainly unclear. A mere altered pharmacokinetic profile does not necessarily mean altered clinical outcome.

For example, in obsess adults (81) Abernethy et al reported a substantial increase in the plasma concentration of verapamil with a three-fold prolongation of terminal half-life but no increase in measured effects. Indeed, there was an increased EC50 in obese patients to prolong P-R interval as compared with controls (45.9 ± 6.7 vs. 22.6 ± 2.0 ng/mL; *p* < 0.005). The authors speculated a “decreased direct verapamil effect and/or impaired baroreflex sensitivity, sympathetic, and parasympathetic reflex responses due to verapamil-induced hypotension in obese as compared to normal-weight hypertensive patients” for their observation (81). However, a down-regulation of the calcium channel receptor target protein, as has been shown in other inflammatory conditions, is likely responsible for this observation.

**FIGURE 9:**
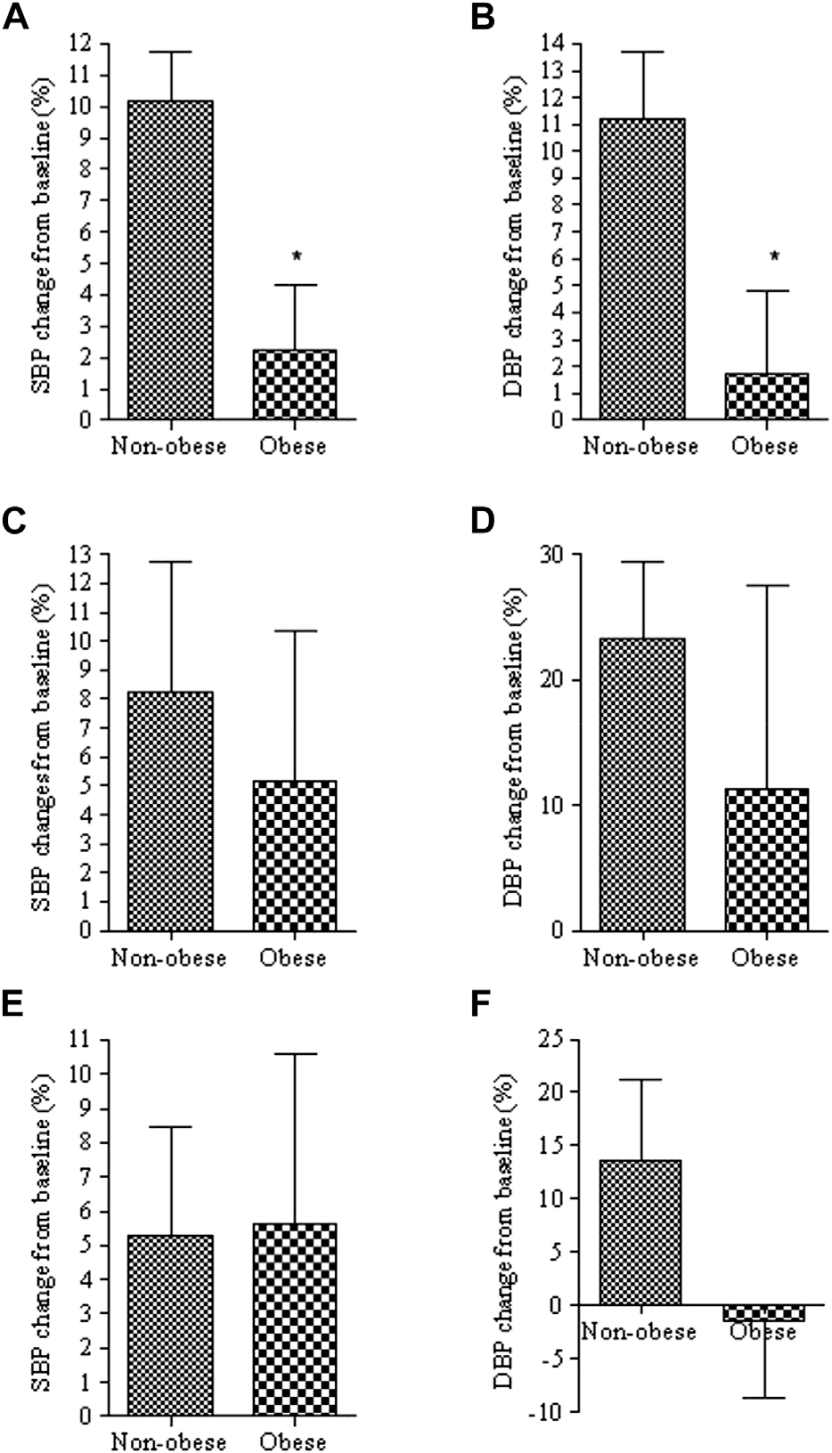
Percent changes of systolic (SBP) and diastolic (DBP) blood pressure from baseline in obese and non-obese patients treated with calcium channel blockers **(A**,**B)**, angiotensin blockers **(C**,**D)** or combination of the two **(E**,**F)**. *, *p* < 0.05 from non-obese. From (132).

The above findings are in concert with another set of data generated bu studying obese children. The study has illustrated a significant reduction in the efficacy of calcium channel blockers used to control blood pressure (132). The study’s goal was to demonstrate a 10% reduction from the baseline systolic BP after administering calcium channel blockers. The number of patients that achieved this outcome was significantly lower in the obese patients (12.5%) than in the non-obese group (52.9%) (132) (Figure 9). Drug exposure had not been measured in these patients, but it is reasonable to assume that it has not been reduced, but likely increased in this population. Thus, the reduced effect in these patients is unlikely to be due to reduced concentration.

### HIV

HIV is characterized as an inflammatory disease due to the presence of elevated cytokines, commonly IL-6, and the increased activity of T lymphocytes continuously combating the virus (133). Many drug regimens rely on the inhibition of CYP enzymes with protease inhibitors such as ritonavir, to maximize pharmacological drug cocktail efficacy. A small human study has shown that, compared with healthy controls, ten HIV-infected men and women had 18% lower hepatic CYP3A activity and 90% lower CYP2D6 activity (134). Additionally, another study has noted that the more poorly controlled HIV, the higher would be the cytokine levels (135). Moreover, one study looked at inflammation-mediated modulation in apparent clearance of atazanavir (136). An inverse correlation has been observed between rising bilirubin secondary to the inhibition of UDP glucuronosyltransferase mediated by atazanavir, and CRP, IL-6, as well as TNF-α- indicating an anti-inflammatory effect mediated by drug therapy. Although these studies provide an introductory insight on CYP modulation in HIV, the results are confounded by the anti-inflammatory effects of the pharmacological therapy thus, more research is needed to understand the impact of the cytokines, and the effect on the antiretroviral pharmacokinetics and therapy outcomes.

### COVID-19 and influenzas

During infections with influenza, and COVID-19, the body employs the innate and adaptive immune system to combat the virus. This, in turn, results in the rise of inflammatory cytokines and the establishment of a systemic inflammatory response. Indeed, anti-inflammatory therapy is an essential part of treatment of infected patients (137, 138). The associated inflammation impacts the metabolism of certain medications such as theophylline used in asthmatic kids when infected with influenza B. Children with a CRP above >0.5 mg/d developed an increase in theophylline toxicity, along with a decline in theophylline clearance, attributed to a downregulation of CYP1A2 (139, 140).

COVID-19 is associated with complications such as thromboembolic events, superimposing bacterial infections, and respiratory distress that contribute to poor outcomes if not promptly treated with properly dosed antithrombotics and antimicrobials, respectively (141–144). With the novelty and spectrum of severity of COVID-19, and the prominence of inflammation associated with disease progression, the effect on action and disposition of medications should be considered, especially in older adults with multiple comorbidities already on a cocktail of medications, as they are the most affected group (145). During the incubation period of the virus, the immune system generates a multitude of cytokines to eliminate the virus and prevent it from reaching a severe state. In certain individuals, the immune response is weak, allowing viral replication to progress. This failure leads to the recruitment of adaptive immune cells to mitigate the viral load (146). If the dual action of innate and adaptive immune responses fails, a cycle of cytokine recruitment is initiated, leading to a pathogenic positive feedback loop called cytokine storm syndrome (147, 148). This results in hyper-inflammation and damage to tissues as well as a possible alteration in drug response as an influx of cytokines that are known to affect pharmacokinetics of drugs are now substantially increased in tissues involved in metabolism of xenobiotics. Additionally, recent studies propose that the virus binds to ACE2 in the alveoli, a receptor that regulates the conversion of angiotensin 2 (a pro-inflammatory mediator) into its anti-inflammatory metabolite, angiotensin 1-7. This occupation of ACE2 results in its depletion in the lungs, causing an imbalance in the inflammatory mediators (elevated angiotensin 2) - leading to greater inflammation in the area, and pulmonary distress (149). Losartan, an ACE2 receptor blocker, affected the SARS-CoV-2 replication of Vero E6 cells *in vitro* (154).

Aside from respiratory distress, extrapulmonary effects such as hepatic manifestations have been reported (146, 149). The infection is accounted as being like a sepsis-like inflammation due to its ability to manifest cytokine storms because of the immune system’s hyperactivity. This feedback loop illustrates the effects cytokines can impose on CYP metabolism, alteration in target receptors, and changes in drug transporters. Cytokines and other inflammatory mediators can have critical implications on the metabolism of drugs used for symptom control. The currently approved drugs for the treatment of COVID-19 are substrates for CYP2C8, CYP2D6, and CYP3A4 enzymes, as well as OATP1B1, and P-glycoprotein drug transporters (P-gp) (150). Remdesivir is also a weak inhibitor of CYP3A4, OATP1B1, and OATP1B3. Based on previous data on the effects of inflammation on these proteins, the impacted metabolism, and the affected efficacy of the drug could have significant consequences in terms of drug related toxicity, subtherapeutic activity of the drug, and impaired clearance within the patient.

Inflammation’s impact on COVID-19 therapy has been observed in some recent studies involving the use of the HIV drug lopinavir/ritonavir used to treat COVID-19. These studies have found that the concentration of lopinavir in COVID-19 patients has been 3.5-fold higher than HIV patients (102). In COVID patients, a significant positive correlation is found between lopinavir plasma concentrations and CRP values (*r* = 0.37, *p* < 0.001). The measured lopinavir concentration has been reported to be significantly lowered by pre-administration of tocilizumab, due likely to the anti-inflammatory effect of the latter (102).

The effect of severity of inflammation on the action and disposition of the newly approved combination anti-COVID-19 drug, nirmatrelvir plus ritonavir (Paxlovid, Pfizer) also raises concerns over its proper use (*Factors affecting CYP expression* Section). The concentration of the active drug is very likely unpredictable due to the effect of the associated inflammation on the metabolizing enzymes.

COVID-19 initially appeared to spare children with only mild symptoms. However, it is now known that a small portion of children can develop a hyperinflammatory syndrome labeled as pediatric inflammatory multisystem syndrome, or cytokine storm. The effect of such an exaggerated expression of cytokines on pharmacotherapy is not known, but there is no reason to believe that it is negative (*Age related considerations* section).

### Age related considerations

In addition to the influence of inflammation on controlling blood pressure secondary to childhood obesity [*Obesity* section, Figure 9 (132)], there are some reports on the impact of inflammation on CYP metabolism in children. These reports are typically limited to acute care conditions such as the asthmatic children affected by Influenza B with small enrollment populations (22, 140). They have, nevertheless, provided some preliminary data regarding the effect of chronic inflammation on the action and disposition of drugs. One study with a sizable number of participants took data from 83 critically ill intensive care unit patients (2 months–17 years old) and created a pharmacokinetic model from the data to predict the alterations in drug metabolism potentially mediated by elevated cytokines (151). The study model illustrated that these patients might have experienced drug related toxicity due to prolonged and elevated drug concentration caused by CYP3A suppression. This suppression, the study proposes, is likely due to the presence of elevated CRP and IL-6 as the model showed the plasma midazolam concentration has been reported to be 2.7-fold higher at a CRP level of 300 mg/L compared to patients with CRP level of 10 mg/L (151).

Another pediatric study analyzed the general CYP expression of 51 children with sepsis, and 6 children with organ failure. The study found that, using antipyrine clearance as a marker, the children with sepsis had a 2-fold decline in CYP expression, and a 4-fold decline in the organ failure patients compared to controls (140). In patients with cystic fibrosis (CF), there is an elevation of neutrophils and increased concentrations of pro-inflammatory mediators in the airways. In another report, when the unbound fraction of drugs was assessed, CF patients did not differ significantly from healthy patients, and that changes in hepatic metabolic activity was selective in patients with CF (152).

It appears that childhood is not a risk factor for the imbalances due to inflammation. However, the above available data suggests that children are not exempt from inflammation induced altered drug action and disposition. In the meantime, Kawasaki syndrome and the recently reported COVID-related cytokine storm cases mainly involve children (153) which is bound to have consequences on the pharmacotherapy on account of increased cytokine expression.

**FIGURE 10:**
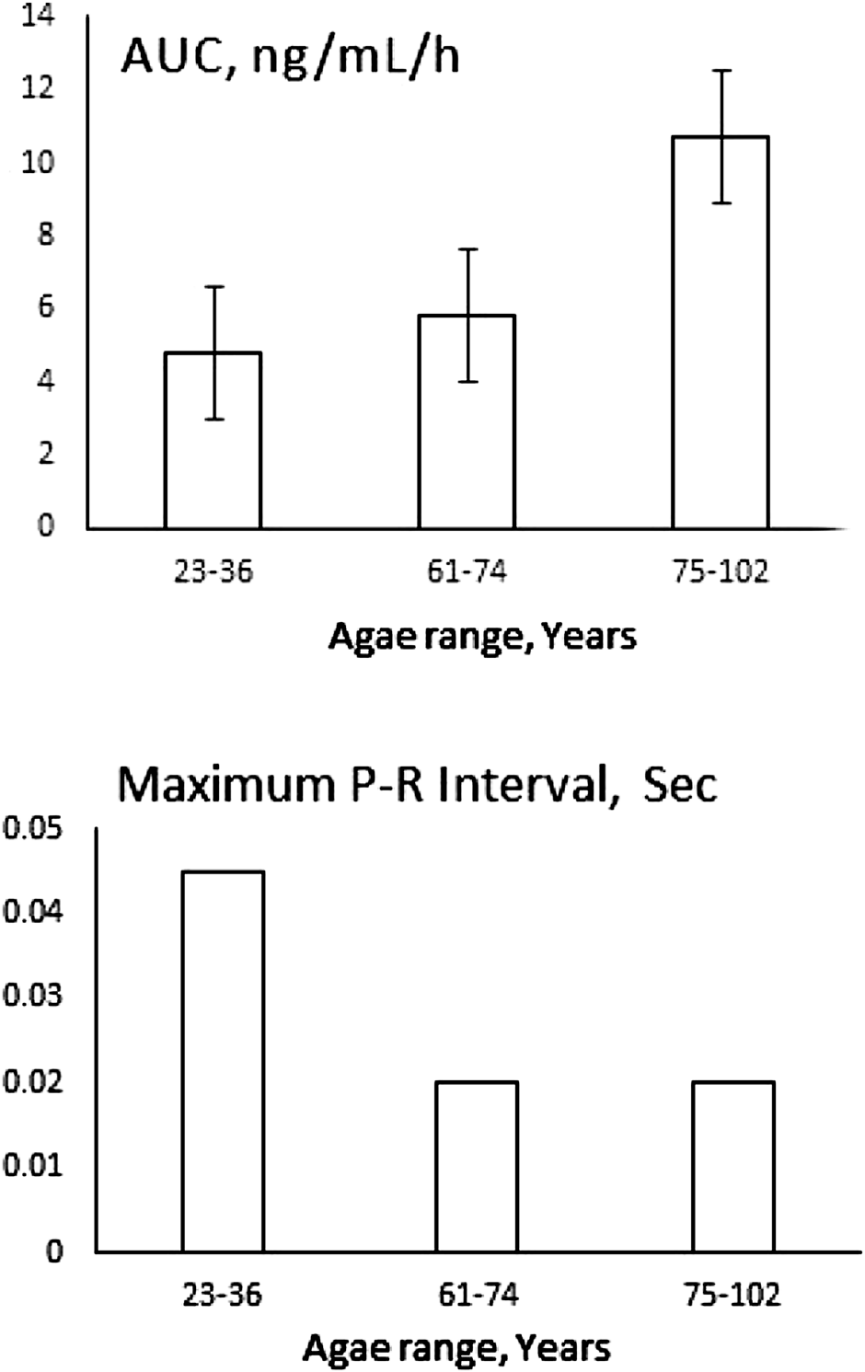
Unexpected reduced effect of 120 mg verapamil on P-R interval of elderly patients despite increased exposure measured as area under plasma concentration-time curve (AUC). Data collected from (81).

Old age also is associated with inflammation (155), and the effect of such a chronic systemic change on pharmacotherapy, although has long been alarmed (156) is often overlooked. Studies mainly focus on pharmacokinetics with no pharmacodynamic data, thus extrapolating plasma concentrations to clinical outcomes. This is while with age the need for pharmacotherapy substantially increases. Through a rare study that included both pharmacokinetics and pharmacodynamics, Abernethy et al. (81) have shown that verapamil clearance is slowed down by aging resulting in increased drug concentration. However, similar to what has been discussed regarding patients with RA, Crohn’s disease and obesity, the effect of the drug to prolong P-R interval had diminished despite increased concentration (Figure 10). The blood pressure lowering effect of the drug was not significantly different between the elderly and young subjects despite different exposures. At the time, the authors were not able to explain their unexpected disconnect between pharmacokinetics and pharmacodynamics observation, The later data, however, is suggestive of simultaneous down regulation of proteins involved in metabolism and drug-receptor interaction. However, the severity of inflammation must be considered for choosing the right cardiovascular therapy regimen for elderly patients.

### Bechet’s disease

Bechet’s disease is characterized as a chronic inflammatory disorder that causes blood vessel inflammation throughout the body, typically controlled by colchicine (157). Goktas et al. compared 52 Bechet’s disease patients with 96 healthy volunteers to assess the genotype and phenotype of CYP2C9 by allele-specific PCR and delineating the ratio of losartan/metabolite ratio, respectively. They found a higher losartan/metabolite ratio in Bechet’s disease as compared with healthy subjects (1.75 vs. 1.02) suggestive of lower CYP2C9 activity in patients. The study ruled out CYP2C9 polymorphisms (157). The therapeutic outcomes of supressed CYPs expression on pharmacotherapy of drugs used to treat non-Bechet’s disease conditions of these patients is unknown mainly due to the scarcity of data stemming from unavailability of sufficient patients for clinical trials.

### Clozapine case reports

The pharmacodynamic impact of clozapine therapy during inflammation has not been well elucidated, however there have been case reports regarding toxicity seen with olanzapine and clozapine in patients with systemic inflammation (158, 159). In one case, a 54-year-old male, who was stabilized on olanzapine injections, developed post-injection delirium/sedation syndrome after taking his seventh olanzapine injection. He was brought into the emergency department and diagnosed with pharyngitis and bronchitis with a CRP rise from 18 to 179 mg/L after 48 h. This toxicity is believed to be mediated by altered metabolism of olanzapine *via* CYP1A1 suppression during the state of inflammation (160).

A similar observation has been made in four patients on clozapine who were admitted to hospital for clozapine related toxicity. Two of the cases had a hypersensitivity reaction to clozapine, while the other two had pneumonia. This resulted in the development of flu-like symptoms and clozapine related toxicities. All of the 4 cases had supratherapeutic clozapine plasma concentrations. The authors proposed that CYP modulation could be a source of toxicity due to clozapine’s influences on the plasma levels of several proinflammatory cytokines such as IL-6 and IL-8 and the inflammatory state that the patients had, potentially impacting CYP1A2 activity, the main clozapine metabolizing enzyme (159).

Another case presents a 23-year-old with schizophrenia admitted to the hospital for a gastrointestinal infection had a trough serum concentration of clozapine at admission of 9074 nmol/L, almost 4-fold the upper limit of the reference range. The patient however did not demonstrate any clozapine-related toxicity (159). The author proposed that the phenomenon was probably due to 1) a downregulation of CYP enzyme activities which primarily seems to be mediated by IL-6, during infection and inflammation and/or 2) an increase in AAG during infection and inflammation.

## Conclusion

Many diseases and conditions influence pharmacotherapy through their association with inflammation. The effect of inflammation is not limited to altered pharmacokinetics since it also influences pharmacodynamics. Relying on pharmacokinetics without solid evidence of a meaningful relationship with pharmacodynamic may result in misleading interpretations. Drug interaction books are populated with alerts solely based on pharmacokinetics data often with no therapeutic data to back the claims. Many pharmacokinetic changes are due to altered expression of proteins responsible for the clearance of drugs. However, there is no reason to believe that a change in the expression of one protein is not more generalizable to cover proteins that are responsible for drug receptor interactions as we have shown in this review. We have covered a limited number of observations in which either there is a disconnect between pharmacokinetics and pharmacodynamics or the therapeutic outcome of the data is unknown. However, this limitation is due to the scarcity of information. It is reasonable to suggest that these drug-disease interactions are not limited to the small example we have covered. The knowledge of the fact that for some drugs, there is no readily understandable concentration-effect relationship although not new, is often ignored. Thus, for any pharmacokinetics and drug metabolism observation, the therapeutic rationale must be considered. This is particularly essential when the drug action and/or disposition is dependent upon the patient condition. For example, as it has clearly been demonstrated, the effect of the condition and the severity of the disease, i.e., rheumatoid arthritis only results in drug-disease interaction when the disease is in its active stage.

## Abbreviations

AA, adjuvant arthritis; ABC, ATP-binding cassette; AAG, Alpha 1 acid glycoprotein; APC, antigen-presenting cells; CYP, cytochrome P450 enzyme; AUC, area under the curve; CAR, constitutive androstane receptor; CD, Crohn’s disease; CF, cystic fibrosis; CRP, C- reactive protein; COVID-19/COVID, coronavirus -19; CVD, cardiovascular disease; DME, drug metabolizing enzyme; GH, growth hormone; HIV, human Immunodeficiency viruses; IL, interleukin; LPS, lipopolysaccharide; NMO, neuromyelitis Optica; NFKB, nuclear factor kappa B; PXR, pregnane X receptor; RA, rheumatoid arthritis; RAS, renin angiotensin system; RXR, retinoid X receptor; SLC, solute carrier; TH, T-Helper; TNF, tumor necrosis factor; UC, ulcerative colitis.
